# The Phosphoinositide 3-Kinase Signaling Pathway is Involved in the Control of Modified Low-Density Lipoprotein Uptake by Human Macrophages

**DOI:** 10.1007/s11745-015-3993-0

**Published:** 2015-02-08

**Authors:** Daryn R. Michael, Thomas S. Davies, Lucia Laubertová, Hayley Gallagher, Dipak P. Ramji

**Affiliations:** 1 Cardiff School of Biosciences, Cardiff University, Sir Martin Evans Building, Museum Avenue, Cardiff, CF10 3AX UK; 2 Institute of Medical Biochemistry, Jessenius Faculty of Medicine, Comenius University, Malá Hora 4, 036 01 Martin, Slovakia

**Keywords:** Atherosclerosis, Foam cell, Cholesterol, Macrophage, Phosphoinositide 3-kinase

## Abstract

The transformation of macrophages into lipid-loaded foam cells is a critical early event in the pathogenesis of atherosclerosis. Both receptor-mediated uptake of modified LDL, mediated primarily by scavenger receptors-A (SR-A) and CD36 along with other proteins such as lipoprotein lipase (LPL), and macropinocytosis contribute to macrophage foam cell formation. The signaling pathways that are involved in the control of foam cell formation are not fully understood. In this study, we have investigated the role of phosphoinositide 3-kinase (PI3K) in relation to foam cell formation in human macrophages. The pan PI3K inhibitor LY294002 attenuated the uptake of modified LDL and macropinocytosis, as measured by Lucifer Yellow uptake, by human macrophages. In addition, the expression of SR-A, CD36 and LPL was attenuated by LY294002. The use of isoform-selective PI3K inhibitors showed that PI3K-β, -γ and -δ were all required for the expression of SR-A and CD36 whereas only PI3K-γ was necessary in the case of LPL. These studies reveal a pivotal role of PI3K in the control of macrophage foam cell formation and provide further evidence for their potential as therapeutic target against atherosclerosis.

**Electronic supplementary material:**

The online version of this article (doi:10.1007/s11745-015-3993-0) contains supplementary material, which is available to authorized users.

## Introduction

Atherosclerosis, the underlying cause of myocardial infarction and stroke, is responsible for most deaths in western society [1]. The disease is initiated by the activation of the arterial endothelium by various risk factors, such as LDL cholesterol and hypertension, which results in the secretion of chemokines, such as monocyte chemotactic protein-1, by endothelial cells [1, 2]. These attract immune cells, particularly T-lymphocytes and monocytes, in a process that is aided by increased expression of adhesion molecules on the endothelial cell surface [1, 2]. The recruited monocytes differentiate into macrophages, which can then take up modified lipoproteins to form lipid-laden foam cells [1, 2]. As the disease progresses, complex fibrotic plaques are formed due to the combined action of foam cell lysis, defective efferocytosis, migration and proliferation of smooth muscle cells from the media to the intima, and synthesis of extracellular matrix by them [1, 2]. Rupture of such plaques by proteolytic enzymes produced by macrophages as part of a continued inflammatory response leads to thrombosis and subsequent clinical complications of this disease [1, 2].

The transformation of macrophages into foam cells is a critical early event in the pathogenesis of atherosclerosis [1, 2]. The uptake of modified LDL by a receptor-mediated mechanism via increased expression of key genes, such as scavenger receptor-A (SR-A) and CD36, by the invading macrophages represents a major mechanism [1, 2]. In addition, the lipoprotein lipase (LPL) expressed by macrophages contributes to foam cell formation via a so-called “bridging action” that leads to the accumulation of modified lipoproteins at the cell surface and their subsequent uptake by the cells [3, 4]. Receptor-independent mechanisms, particularly macropinocytosis, also play an important role in foam cell formation [5, 6]. Macropinocytosis is a form of fluid-phase endocytosis where the uptake of solute does not reach saturation when its concentration is increased (i.e. solute uptake is directly proportional to its concentration) [5, 6]. The process involves actin-dependent ruffling of the plasma membrane and the subsequent fusion of the membrane to itself to form intracellular fluid filled vacuoles 0.5–5 μM in diameter [5, 6]. Although an important role for macropinocytosis in the uptake of native LDL is well established [5, 6], our recent studies have extended the role of this process to the uptake of modified LDL by macrophages [7, 8]. Previous studies have shown that PI3K plays a potentially important role in macropinocytosis, including the uptake of native LDL, under certain conditions [5, 6]. However, the role of PI3K in the uptake of modified LDL and the expression of key genes implicated in this process is not fully understood, and formed the focus of the current study.

## Materials and Methods

### Reagents

LY294002 was obtained from Merck Millipore (Calbiochem) whereas isoform-specific inhibitors TGX-221, IC-87114 and AS-605240 were from Selleck Chemicals. 1,1′-Dioctadecyl-3,3,3′,3′-tetramethyllindocarbocyane perchlorate (DiI)-labeled acetylated LDL (DiI-AcLDL) was purchased from Intracel. All the other chemicals were obtained from Sigma-Aldrich unless otherwise stated.

### Cell Culture

Human monocyte-derived macrophages (HMDM) were differentiated from monocytes isolated from buffy coats from the Welsh Blood Service using Ficoll-Hypaque purification as previously described [7–10]. The human monocytic leukemia THP-1 cell line and HMDM were grown in RPMI-1640 supplemented with 10 % (v/v) heat-inactivated fetal calf serum, penicillin (100 U/ml), streptomycin (100 μg/ml) and l-glutamine (2 mmol/l) (Invitrogen) at 37 °C in a humidified atmosphere containing 5 % (v/v) CO_2_. The THP-1 monocytes were differentiated into macrophages using 160 nM of PMA for 24 h, a period corresponding to high levels of expression of scavenger receptors and other key genes implicated in the regulation of macrophage foam cell formation [7–12].

### DiI-AcLDL and Lucifer Yellow Uptake Assays

THP-1 macrophages were treated for 24 h at 37 °C with 10 μg/ml DiI-AcLDL or 100 μg/ml Lucifer Yellow (LY) (concentrations of both based on previous studies) in RPMI-1640 containing 0.2 % (v/v) fatty-acid free BSA [8–10]. The uptake was analyzed by flow cytometry on a FACS Canto (BD Biosciences, Oxford, UK) flow cytometer with 10,000 events acquired for each sample. The uptake was represented as a percentage with the vehicle-treated control arbitrarily assigned as 100 %.

### Real-Time Quantitative PCR

The extraction of RNA, reverse transcription and real-time quantitative PCR (RT-qPCR) of the genes analyzed was carried out as previously described [7–10]. The sequences of the primers were: 5′-CCAGGGACATGGAATGCAA-3′ and 5′-CCAGTGGGACCTCGATCTCC-3′ for SR-A [13]; 5′-GAGAACTGTTATGGGGCTAT-3′ and 5′-TTCAACTGGAGAGGCAAAGG-3′ for CD36 [13]; 5′-GAGATTTCTCTGTATGGCACC-3′ and 5′-CTGCAAATGAGACACTTTCTC-3′ for LPL [14]; and 5′-CCTGGAGGAGAAGAGGAAAGAGA-3′ and 5′-TTGAGGACCTCTGTGTATTTGTCAA-3′ for 60S ribosomal protein L13a (RPL13A). The fold changes in expression were determined using $2^{{ - \left( {\Delta C_{{{\text{t}}_{1} }} - \Delta C_{{{\text{t}}_{2} }} } \right)}}$, where Δ*C*_t_ represents the difference between the threshold cycle (*C*_t_) for each target gene and RPL13A mRNA transcript levels.

### Western Blot Analysis

Total cell lysates were size-fractionated on SDS-polyacrylamide gels (Life Technologies) and then analyzed by Western blot analysis as previously described [7, 9, 10]. Antibodies were either from Santacruz Biotechnology [SR-A (sc-20660), CD36 (sc-9154)] or Sigma-Aldrich (β-actin). Semi-quantitative measurement of signals in Western blots was performed by densitometric analysis using the Image J software.

### Statistical Analysis

The Student’s *t* test was used for single comparisons. For multiple comparisons, one-way ANOVA with Tukey’s or Games-Howell post hoc analysis was used. Values of *P* were considered significant below 0.05.

## Results

### The Uptake of Modified LDL by Human Macrophages is Attenuated by the PI3K Inhibitor LY294002

**Fig. 1:**
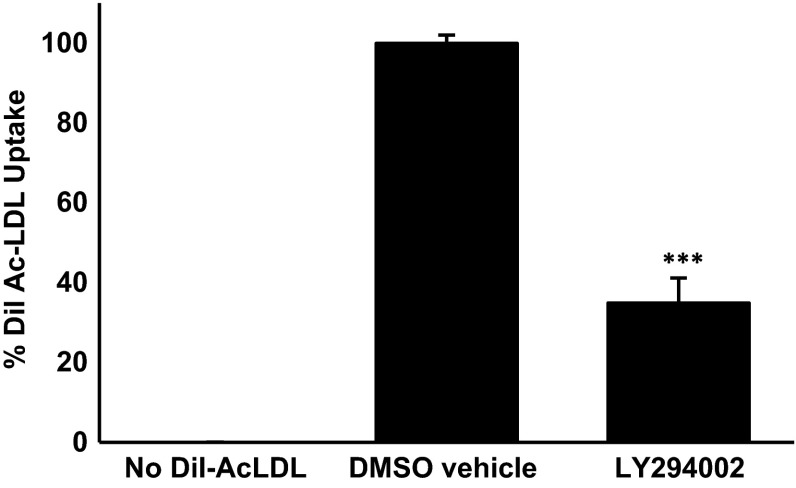
LY294002 attenuates AcLDL uptake by THP-1 macrophages. The uptake of DiI-AcLDL was determined in response to 24 h of incubation with DMSO vehicle control or 100 μM LY294002. Data represent means ± SD from four independent experiments, the uptake in vehicle treated cells has been arbitrarily assigned as 100 %. Statistical analysis was performed using the Student’s *t* test, ***P < 0.001

PMA differentiated THP-1 macrophages are widely used as a model system to investigate the regulation of macrophage function and gene expression in relation to atherosclerosis with demonstrated conservation of responses to primary cultures and in vivo [7–12, 14]. This model system was used initially to investigate the uptake of AcLDL, which is widely used for such assays because the cells avidly take them up and shows excellent correlation with the uptake of oxidized LDL [7–10, 15, 16]. The effect of the pan PI3K inhibitor, LY294002, on AcLDL uptake by THP-1 macrophages was first investigated. As shown in Fig. 1, the inhibitor produced a statistically significant inhibition of AcLDL uptake of about 60 %. The concentration of LY294002 used in these initial experiments was 100 μM and hence it was decided to carry out a dose response experiment. The reduction in AcLDL uptake was observed with 10 μM LY294002 with maximal decrease at the 100 μM concentration (Supplementary Figure 1).

**Fig. 2:**
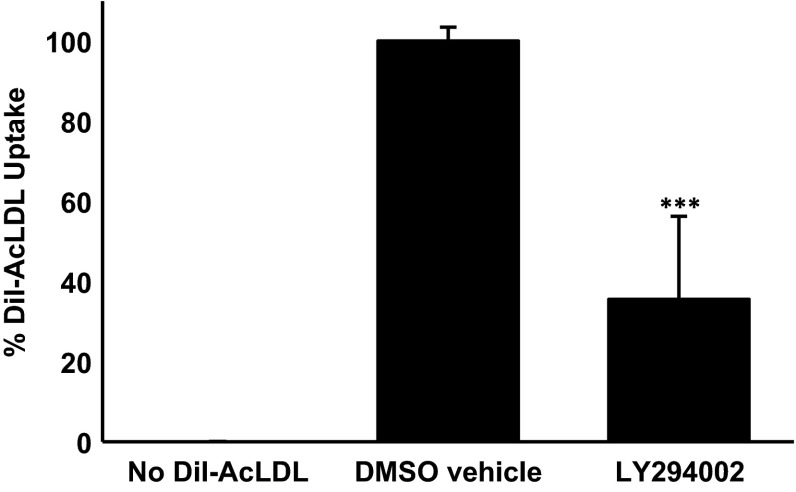
LY294002 attenuates AcLDL uptake by HMDM. The uptake of DiI-AcLDL was determined in response to 24 h incubation with DMSO vehicle control or 100 μM LY294002. Data represent means ± SD from three independent experiments, the uptake in vehicle treated cells has been arbitrarily assigned as 100 %. Statistical analysis was performed using the Student’s *t* test, ***P < 0.001

In order to rule out the possibility that the inhibition of AcLDL uptake by LY294002 was peculiar to the THP-1 cell line, representative experiments were carried out on primary cultures of HMDM. As shown in Fig. 2, LY294002 also inhibited the uptake of AcLDL by HMDM by approximately 60 %. Thus, LY294002 inhibits AcLDL uptake in both THP-1 macrophages and HMDM.

### LY294002 Inhibits LY Uptake and Macropinocytosis by Human Macrophages

**Fig. 3:**
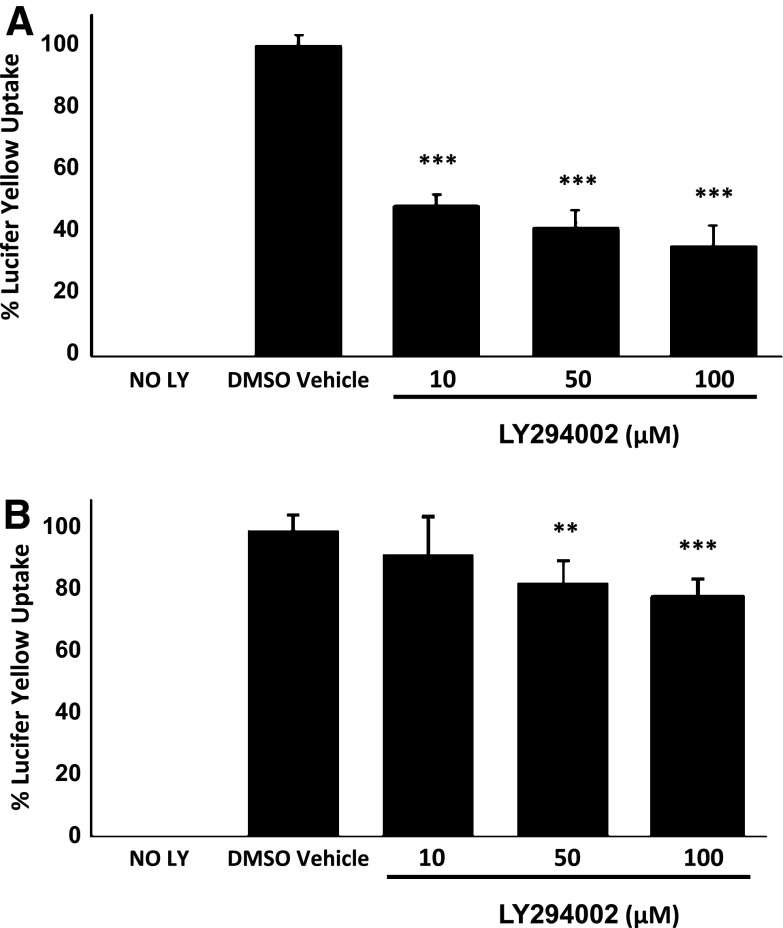
LY294002 inhibits LY uptake in human macrophages. LY uptake was determined in THP-1 macrophages (**a**) or HMDM (**b**) in response to 24 h incubation with DMSO vehicle control or the indicated concentration of LY294002. Data represent means ± SD from three independent experiments, the uptake in vehicle treated cells has been arbitrarily assigned as 100 %. Statistical analysis was performed using one-way ANOVA with Games-Howell (**a**) or Tukey’s (**b**) *post hoc* analysis, ***P < 0.001, **P < 0.01

The fluorescent dye LY is commonly used to monitor macropinocytosis [7, 8, 17]. The action of LY294002 on LY uptake was investigated in THP-1 macrophages and HMDM. Figure 3a shows that LY294002 inhibits the uptake of LY at all the concentrations used (10, 50 and 100 μM). In the case of HMDM, LY294002 had no effect on LY uptake at 10 μM but produced a significant reduction at 50 and 100 μM (Fig. 3b). Thus, a similar trend in the action of LY294002 on the uptake of LY was observed in both THP-1 macrophages and HMDM.

### LY294002 Inhibits the Expression of SR-A, CD36 and LPL in Human Macrophages

**Fig. 4:**
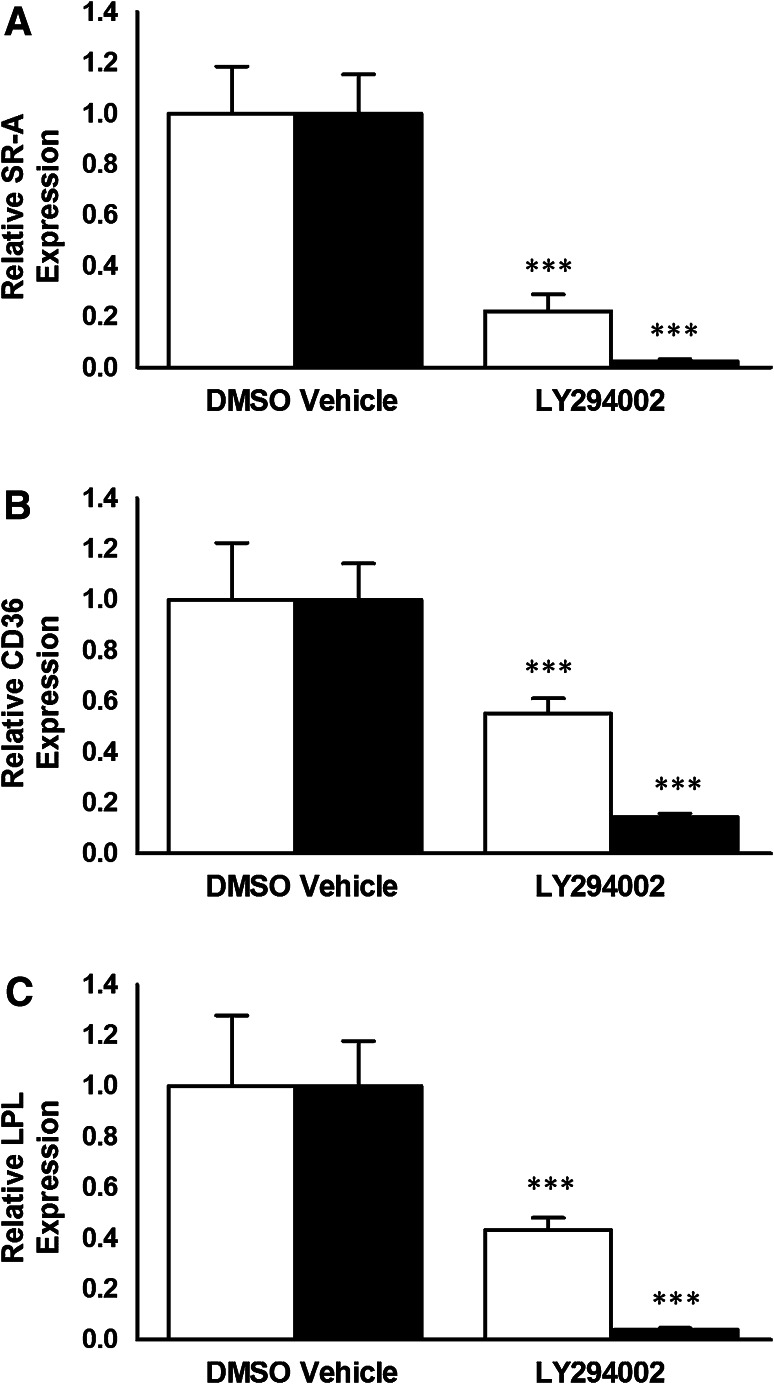
LY294002 inhibits the expression of SR-A, CD36 and LPL mRNA in THP-1 macrophages. THP-1 macrophages were incubated for 24 h with the DMSO vehicle or 10 μM LY294002 (*empty bars*) or 100 μM LY294002 (*filled bars*). Total RNA was subjected to real-time quantitative PCR using primers against SR-A, CD36 or LPL as indicated. The mRNA expression levels were calculated using the comparative *C*_t_ method and normalized to RPL13A with vehicle-treated cells given an arbitrary value of 1. Data represent means ± SD from three (for 100 μM LY294002) or five (for 10 μM LY294002) independent experiments. Statistical analysis was performed using the Student’s *t* test, ***P < 0.001

**Fig. 5:**
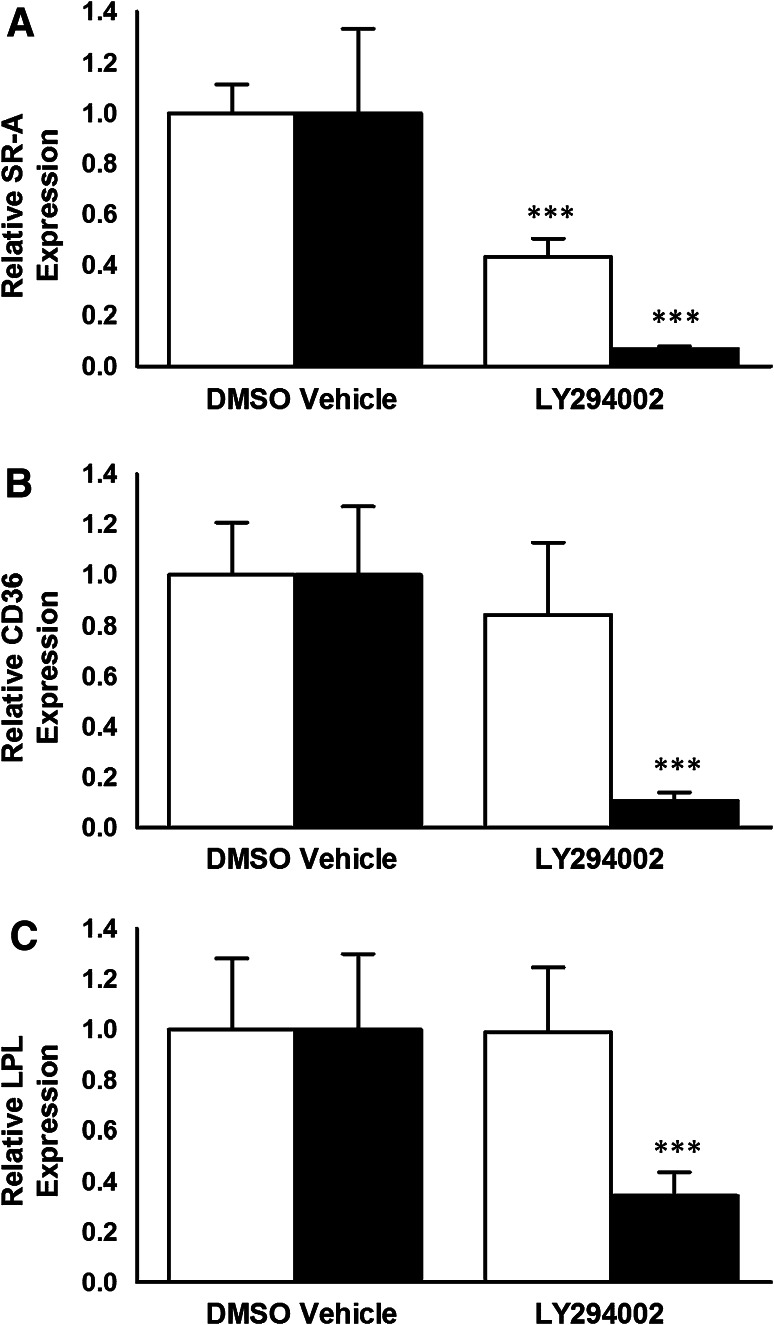
The effect of LY294002 on the expression of SR-A, CD36 and LPL mRNA in HMDM. HMDM were incubated for 24 h with the DMSO vehicle or 10 μM LY294002 (*empty bars*) or 100 μM LY294002 (*filled bars*). Total RNA was subjected to real-time quantitative PCR using primers against SR-A, CD36 or LPL as indicated. The mRNA expression levels were calculated using the comparative *C*_t_ method and normalized to RPL13A with vehicle-treated cells given an arbitrary value of 1. Data represent means ± SD from three independent experiments. Statistical analysis was performed using the Student’s *t* test, ***P < 0.001

The effect of LY294002 on the expression of key genes in macrophages implicated in the uptake of modified LDL was next investigated. LY294002 was used at two different concentrations, 10 and 100 μM. As shown in Fig. 4, LY294002 at both concentrations attenuated the expression of SR-A, CD36 and LPL mRNA in THP-1 macrophages. In the case of HMDM, LY294002 inhibited the mRNA expression of all three genes when used at a concentration of 100 μM though a significant reduction of only SR-A mRNA levels was observed with 10 μM LY294002 (Fig. 5). Thus, a similar trend of action of LY294002 was observed in both THP-1 macrophages and HMDM.

**Fig. 6:**
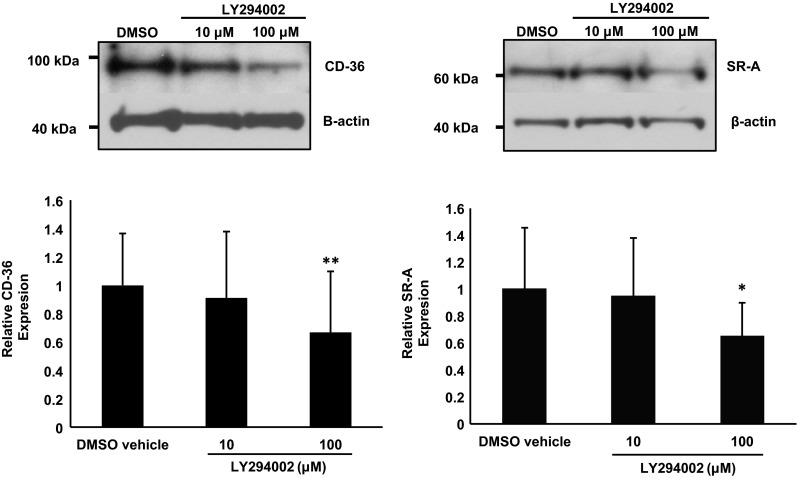
LY294002 inhibits the steady state levels of SR-A and CD36 in THP-1 macrophages. THP-1 macrophages were incubated for 24 h with the DMSO vehicle or 10 or 100 μM LY294002. Equal amount of protein extracts were subjected to Western blot analysis using antisera against SR-A, CD36 or β-actin. The image shown is representative of three independent experiments. The *histograms* show the levels of SR-A or CD36 normalized to β-actin (means ± SD), as determined by densitometric analysis, with the relative expression in DMSO control arbitrarily assigned as 1. Statistical analysis was performed using one-way ANOVA with Tukey’s post hoc analysis, *P < 0.05, **P < 0.01

In order the investigate whether the effect of LY294002 on mRNA expression was also accompanied by corresponding changes in protein levels, Western blot analysis was carried out on SR-A and CD36 in THP-1 macrophages. As shown in Fig. 6, a significant reduction in the steady state levels of SR-A and CD36 was observed with 100 μM LY294002. Thus, LY294002 decreased both mRNA and protein expression of SR-A and CD36.

In the case of RT-qPCR and Western blot analysis, the expression of genes implicated in the uptake of modified LDL (i.e. SR-A, CD36 or LPL) were normalized to a housekeeping gene (RPL13A for RT-qPCR and β-actin for Western blot analysis). These findings suggest that the marked reduction in gene expression is unlikely because of an effect of LY294002 on cell viability. Nevertheless, representative experiments on cell viability were carried out using the crystal violet assay. As shown in Supplementary Figure 2, LY294002 had no significant effect on the viability of HMDM at all concentrations used. A slight but significant reduction in the viability of THP-1 macrophages was observed with 50 and 100 μM LY294002. Overall, these results confirm that the marked action of LY294002 on gene expression cannot be attributed to changes in cell viability as inhibition of gene expression was observed at concentrations that has no effect on cell viability (i.e. all concentrations in HMDM and 10 μM in THP-1 cells). In addition, the extent of changes in gene expression at 50 and 100 μM LY294002 in THP-1 macrophages was substantially greater than that observed at the level of cell viability.

### More Isoform-Selective Inhibitors of the PI3K Pathway also Attenuate the Expression of SR-A, CD36 and LPL in Human Macrophages

**Fig. 7:**
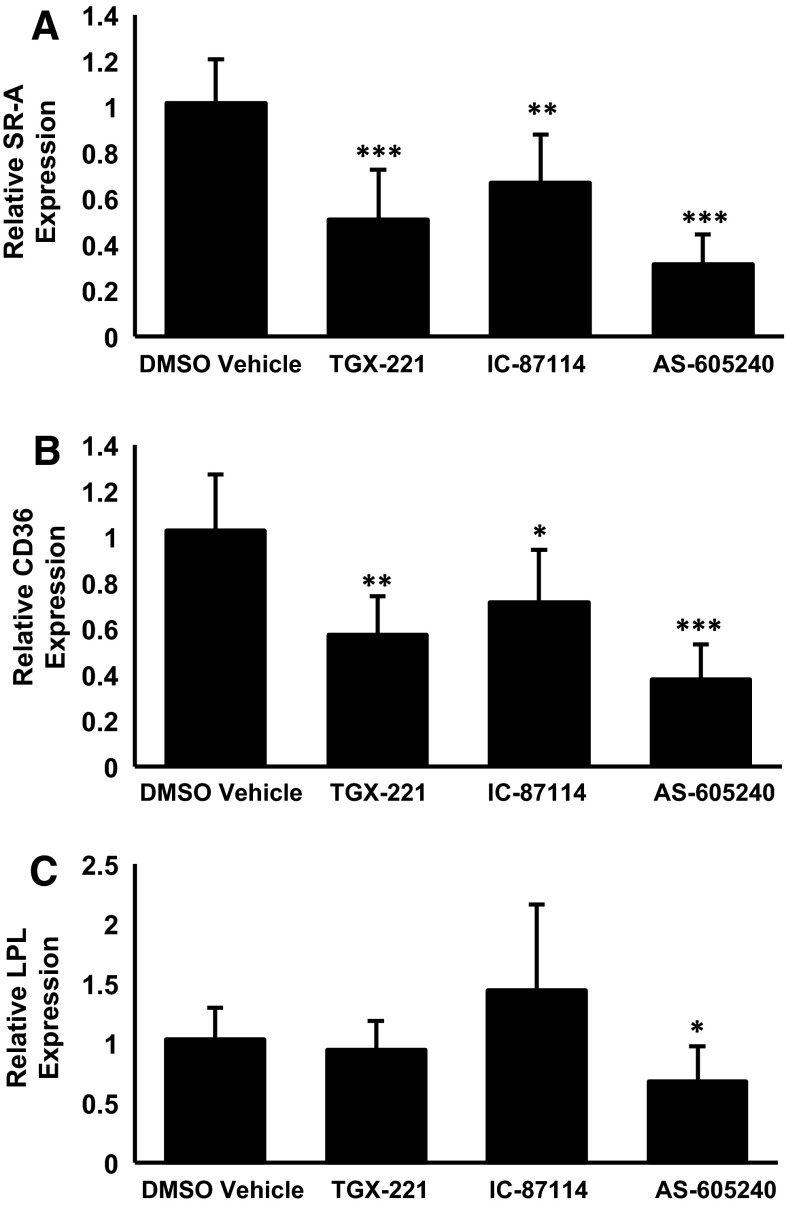
The effect of isoform-specific PI3K inhibitors on the expression of SR-A, CD36 and LPL. THP-1 macrophages were incubated for 24 h with the DMSO vehicle or 10 μM of TGX-221, IC-87114 or AS-605240. Total RNA was subjected to real-time quantitative PCR using primers against **a** SR-A, **b** CD36 or **c** LPL as indicated. The mRNA expression levels were calculated using the comparative *C*_t_ method and normalized to RPL13A with vehicle-treated cells given an arbitrary value of 1. Data represent the means ± SD from four independent experiments. Statistical analysis was performed using one-way ANOVA with Games-Howell post hoc analysis, *P < 0.05, **P < 0.01, ***P < 0.001

In order to further evaluate the importance of PI3K signaling, the action of three isoform-specific inhibitors, TGX-221 (β), IC-87114 (δ) and AS-605240 (γ), on the expression of SR-A, CD36 and LPL mRNA was next investigated. Preliminary dose response experiments using LY294002 as a positive control showed that maximal inhibition, where obtained, was achieved at 10 μM concentration (Supplementary Figure 3) so this concentration was used for subsequent studies. The expression of SR-A and CD36 was significantly attenuated by all three inhibitors, thereby suggesting the role of multiple PI3K isoforms in the regulation (Fig. 7). On the other hand, a significant reduction of LPL was only obtained with the PI3Kγ-specific inhibitor AS-605240 (Fig. 7).

## Discussion

Macrophage foam cell formation is a complex process with receptor-mediated uptake of modified LDL along with other processes such as macropinocytosis playing key roles [1, 2, 5, 6]. The impact of different signaling pathways on macrophage uptake of modified LDL along with associated changes in gene expression is so far poorly understood. We show here that the PI3K signaling pathway is critical for the uptake of modified LDL, macropinocytosis and the expression of key genes implicated in this process.

Receptor-mediated endocytosis plays a key role in the uptake of modified LDL with SR-A and CD36 being important regulators of the process [1, 2]. For example, CD36-dependent signaling cascade was demonstrated to be critical for macrophage foam cell formation [18] and deficiency of SR-A or CD36 was found to reduce atherosclerotic development in mouse model systems [19, 20]. However, not all studies have indicated such a key role at least as far as foam cell formation is concerned [21, 22]. This suggests that either other genes and/or non-receptor-mediated processes contribute to macrophage foam cell formation. The LPL enzyme expressed by macrophages has been shown to contribute to foam cell formation because of its ability to bind to both the cell surface and lipoproteins via different regions that cause the accumulation of the latter and their subsequent uptake by the cells (bridging action) [3, 4]. Interestingly, our previous studies had shown that PI3K is involved, at least in part, in the IFN-γ-mediated regulation of LPL expression [23] and this study extends this to its constitutive expression in human macrophages. In addition to receptor-mediated uptake of LDL, fluid-phase pinocytosis, particularly macropinocytosis, has recently been found to play an important role in the control of macrophage foam cell formation [5, 6]. Previous studies on the importance of macropinocytosis were restricted to LDL [5, 6, 24, 25] but we found that this also extends to modified LDL [7]. Overall, our studies demonstrate that PI3K is important for both receptor-mediated uptake processes and macropinocytosis, and confirms links that were identified in some previous studies [5, 6, 24–26].

The PI3K family is complex with at least three distinct classes, some with several members, and inhibitors against the catalytic subunits of Class Ia family (p110α, p110β and p110δ) are being developed as therapeutic agents against several aspects of cardiovascular disease [27–29]. In terms of studies on mouse model systems, previous research has been restricted to PI3Kγ whose deletion was found to attenuate the development of atherosclerosis [30, 31]. Interestingly, PI3Kγ was also found to be critical for granulocyte macrophage-colony stimulating factor-differentiated murine macrophages to become foam cells by fluid-phase pinocytosis of LDL [24]. In contrast, fluid-phase pinocytosis of native LDL by macrophage colony stimulating factor-differentiated macrophages was not affected by pharmacological inhibition of all four-class I PI3K isoforms [25]. This suggests that the overall role of PI3K family in the control of macrophage foam cell formation is likely to be complex. Interestingly, our studies also show that whereas specific inhibitors against PI3K-β, -γ and -δ all inhibit the expression of SR-A and CD36, LPL levels are only affected by the PI3Kγ inhibitor (Fig. 7). These data argue against inhibition of a single isoform in the therapeutic intervention of macrophage foam cell formation.

In conclusion, we have demonstrated a pivotal role of PI3K signaling in the uptake of modified LDL, macropinocytosis and the expression of key genes implicated in the control of foam cell formation in human macrophages. Future studies should investigate the molecular mechanisms underlying PI3K actions leading to changes in the expression of key genes implicated in foam cell formation.

## Electronic supplementary material

Below is the link to the electronic supplementary material.

Supplementary material 1 (PDF 155 kb)

## Abbreviations

AcLDL: Acetylated low density lipoprotein
HMDM: Human monocyte-derived macrophages
LDL: Low density lipoprotein
LPL: Lipoprotein lipase
LY: Lucifer Yellow
PI3K: Phosphoinositide 3-kinase
RT-qPCR: Real-time quantitative PCR
SR-A: Scavenger receptor-A
