# Supplementary material for: The Phosphoinositide 3-Kinase Signaling Pathway is Involved in the Control of Modified Low-Density Lipoprotein Uptake by Human Macrophages
Source: Lipids. 2015 Feb 8;50(3):253–60. doi: 10.1007/s11745-015-3993-0 (PMC4339697; doi:10.1007/s11745-015-3993-0)
Supplement: Supplementary file 1 — Supplementary material 1 (PDF 155 kb) [file 11745_2015_3993_MOESM1_ESM.pdf]

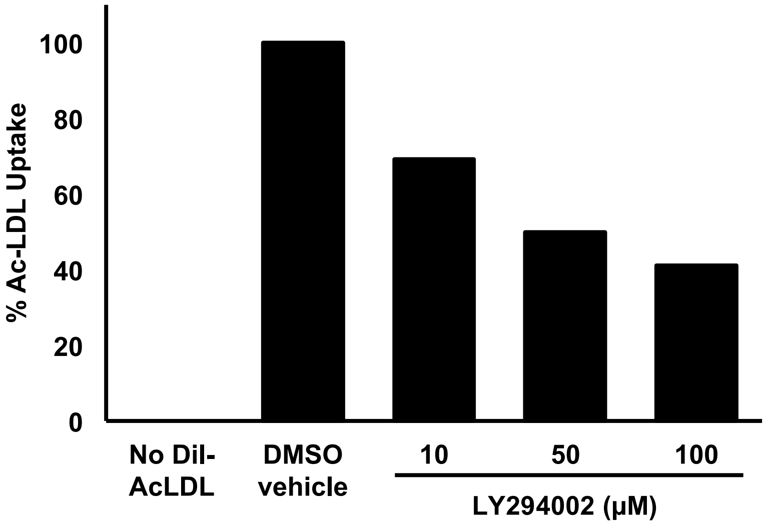


**Supplementary Figure 1. LY294002 attenuates AcLDL uptake by THP-1 macrophages in a concentration dependent manner**

The uptake of DiI-AcLDL was determined in response to 24 h incubation with DMSO vehicle control or the indicated concentration of LY294002. The uptake in vehicle treated cells has been arbitrarily assigned as 100%. Data represents mean of duplicate samples from one experiment.

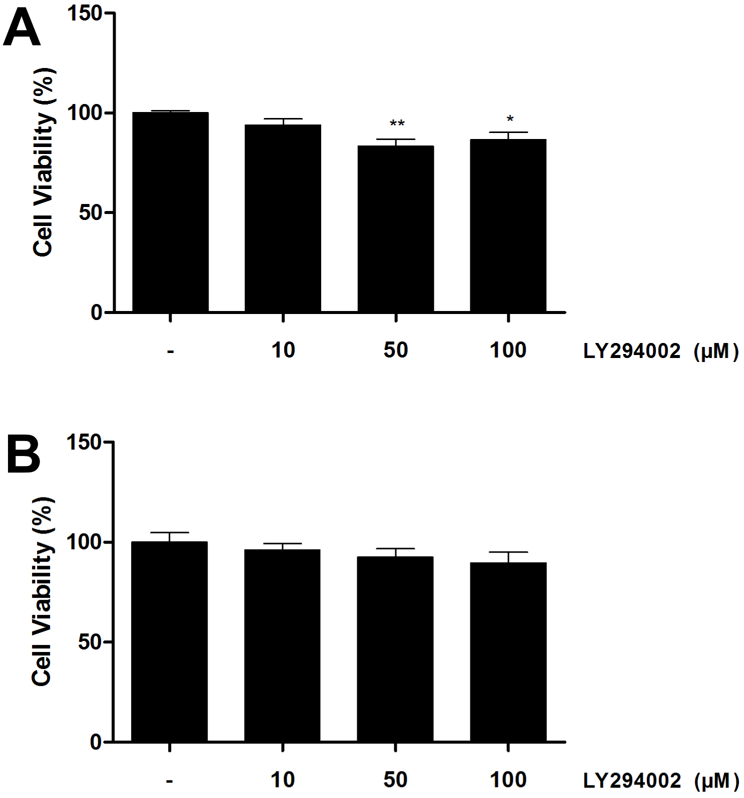


**Supplementary Figure 2. The effect of LY294002 on the viability of THP-1 macrophages and HMDM**

THP-1 macrophages (A) or HMDM (B) were incubated for 24 h with the DMSO vehicle (-) or the indicated concentration of LY294002. Cell viability was determined using crystal violet. Cell viability in the presence of LY294002 (mean ± SD from three independent experiments) is represented to control cells, which has been arbitrarily assigned as 100%. Statistical analysis was performed using one-way ANOVA with Tukey's post-hoc analysis, *$P < 0.05$, **$P < 0.01$.

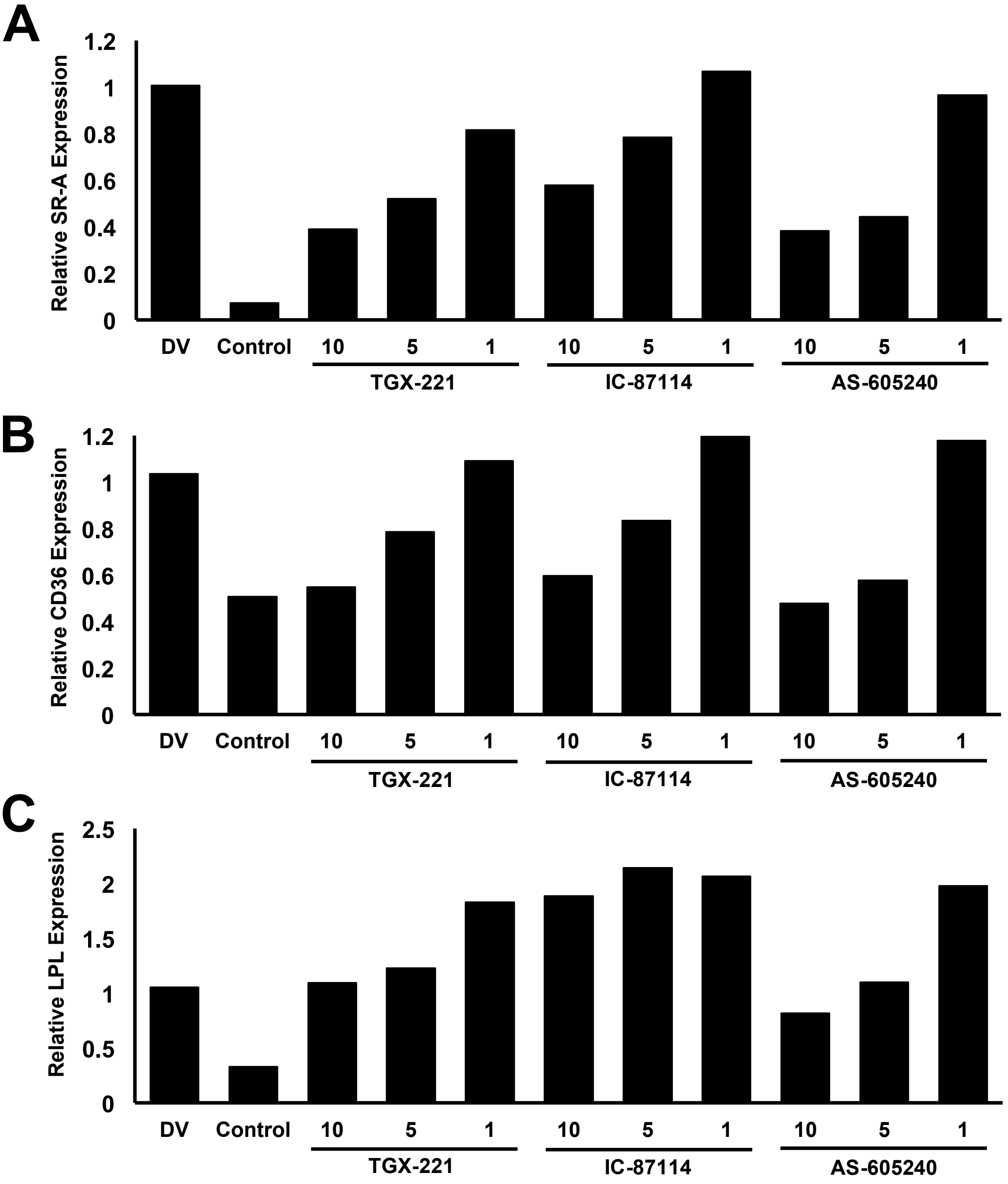


**Supplementary Figure 3. The effect of isoform-specific PI3K inhibitors on the expression of SR-A, CD36 and LPL**

THP-1 macrophages were incubated for 24 h with the DMSO vehicle (DV) or 10 uM LY294002 (control) or the indicated concentration of TGX-221, IC-87114 or AS-605240. Total RNA was subjected to real-time quantitative PCR using primers against (A) SR-A, (B) CD36 or (C) LPL as indicated. The

mRNA expression levels were calculated using the comparative C*t* method and normalized to RPL13A with vehicle-treated cells given an arbitrary value of 1. Data represents the mean of two independent experiments performed with triplicate samples.
